# Evaluation of p16^INK4a^ expression as a single marker to select patients with HPV-driven oropharyngeal cancers for treatment de-escalation

**DOI:** 10.1038/s41416-020-0964-x

**Published:** 2020-07-06

**Authors:** Steffen Wagner, Elena-Sophie Prigge, Nora Wuerdemann, Henrike Reder, Ayman Bushnak, Shachi Jenny Sharma, Theresa Obermueller, Magnus von Knebel Doeberitz, Thomas Dreyer, Stefan Gattenlöhner, Gregor Wolf, Jörn Pons-Kühnemann, Claus Wittekindt, Jens Peter Klussmann

**Affiliations:** 1 Department of Otorhinolaryngology, Head and Neck Surgery, University of Giessen, Giessen, Germany; 2 Department of Applied Tumor Biology, Institute of Pathology, University Hospital Heidelberg, Heidelberg, Germany; 3 Clinical Cooperation Unit Applied Tumor Biology, German Cancer Research Center (DKFZ), Heidelberg, Germany; 4 Department of Otorhinolaryngology, Head and Neck Surgery, Medical Faculty, University of Cologne, Cologne, Germany; 5 Institute of Pathology, University of Giessen, Giessen, Germany; 6 Medical Statistics, Institute of Medical Informatics, University of Giessen, Giessen, Germany

**Keywords:** Prognostic markers, Oral cancer, Tumour biomarkers, Tumour virus infections, Cancer therapy

## Abstract

**Background:**

A remarkably better prognosis is associated with oropharyngeal squamous cell carcinomas (OPSCC) driven by human papillomaviruses (HPV) compared with HPV-negative OPSCC. Consequently, de-escalation of standard treatment has been suggested. Due to modest specificity rates, debates are ongoing, whether p16^INK4a^, a surrogate marker for HPV-driven OPSCC, is sufficient to correctly identify those tumours and avoid substantial HPV misattribution and thus undertreatment of patients by de-escalation. Robust data estimating the proportion of potentially undertreated patients are missing.

**Methods:**

We assessed a large-scale cohort of consecutively included OPSCC diagnosed between 2000 and 2017 for HPV–DNA, HPV genotypes, p16^INK4a^ expression and multiple tumour- and patient-related risk factors, and investigated their impact on patients’ survival in comprehensive uni- and multivariate analyses.

**Results:**

Aetiological relevance of HPV (p16^INK4a^- and high-risk HPV–DNA-positivity) was detected in 27.1% (*n* = 192) of OPSCC, with HPV_16_ being the most abundant HPV type (94.6%). In 5.5% patients (*n* = 39), p16^INK4a^ overexpression but no HPV–DNA was detected. Principal component and survival analyses revealed that 60.6% of these p16^INK4a^-positive OPSCC lacking HPV–DNA did not resemble HPV_16_-driven but HPV-negative OPSCC regarding risk-factor profile and overall survival. Notably, this group represented 10.6% of all p16^INK4a^-overexpressing OPSCC.

**Conclusions:**

p16^INK4a^ as a single marker appears insufficient to indicate OPSCC patients suitable for treatment de-escalation.

## Background

Persistent infections with high-risk (HR) human papillomavirus (HPV) types are causative for about 31% of oropharyngeal squamous cell carcinomas (OPSCC).^1^ Importantly, the incidence of HPV-driven OPSCC has been rising in several countries worldwide,^1–3^ albeit heterogeneity exists regarding anatomic subsite, geography and sex.^4^ Patients with HPV-driven OPSCC are reported to have a different risk profile (younger age, reduced tobacco/alcohol consumption) in comparison with HPV-negative OPSCC.^5,6^ Likewise, patients’ survival is remarkably better, although most patients present with smaller primary tumours, but more advanced cervical lymph node metastasis status compared with HPV-negative OPSCC patients.^5^ In view of the superior survival, several clinical phase I–III trials are currently investigating the benefits of de-escalating treatment for this patient group to spare them treatment-associated morbidity.^7–11^ However, de-escalation by replacement of cisplatin with cetuximab failed in the setting of cisplatin-based chemo–radiotherapy.^12,13^ This situation raises the central question which diagnostic test can reliably identify HPV-driven OPSCC patients suitable for treatment de-escalation.^14^

The detection of viral oncogene E6/E7 mRNA or protein can be considered as gold standard to define an aetiological relevance of HPV for carcinogenesis, since E6 and E7 expression is essential for initiation and maintenance of the transformed phenotype in HPV-induced tumours.^15^ However, the technically rather laborious detection procedure for E6/E7 products generally limits its application in routine laboratory testing. Among the various consequences of HPV E6/E7 oncogene signalling, the cellular cyclin-dependent kinase inhibitor 2A (p16^INK4a^) becomes overexpressed as a sequel to E7-induced epigenetic remodelling.^16^ Thus, p16^INK4a^ has been suggested as a biomarker of HPV-transformed cells, and evaluation of p16^INK4a^ expression can be easily performed by immunohistochemistry using formalin-fixed tissue. Detection of p16^INK4a^ has been introduced as a surrogate marker in the AJCC-8/UICC-8 staging system for the classification of OPSCC.

Several studies, however, have demonstrated a lack of association between HPV–DNA or RNA detection and p16^INK4a^ overexpression in about 5–20% of OPSCC, indicating that a considerable subset of p16^INK4a^-positive OPSCC is not causally driven by HPV.^14,17–19^ This fact has raised concerns whether a group of OPSCC patients could be undertreated if the decision for therapy de-escalation was solely based on p16^INK4a^ positivity.^14^ However, there is currently a paucity of robust data estimating the proportion of potentially undertreated patients in this context.

The major goal of our study was to determine the proportion of potentially undertreated patients if p16^INK4a^ represented the single-decision criterion for treatment de-escalation in OPSCC patients. We therefore assessed p16^INK4a^, HPV–DNA and HPV genotypes in relation to tumour- and patient-related risk factors and survival in a cohort of more than 800 consecutively included OPSCC patients at a large German University Hospital diagnosed between 2000 and 2017. Specifically, we aimed to determine risk groups among OPSCC with discordant test results for p16^INK4a^ expression and HPV–DNA to assess whether the respective patient groups could be considered suitable for treatment de-escalation.

## Methods

### Patients and treatment

Between 2000 and 2017, 802 patients were diagnosed with OPSCC (according to ICD10) and treated at our hospital (Department of Otorhinolaryngology, Head and Neck Surgery of the University Hospital Giessen) by upfront surgery with or without neck dissection, or by definitive radio- or chemo–radiotherapy according to the local guidelines and upon patient’s decision (Table 1). Written informed consent was obtained from all patients, and tumour material was used in accordance with the local ethics committee. Those not treated with curative intent were not included in the analysis regarding survival.

**Table 1:** Descriptive analysis of biometric data and treatment of OPSCC patients with concordant HPV–DNA and p16^INK4a^ tests included in the analysis (*N* = 620).

	All	HPV-negative	HPV_16_-driven	*P* value	p16^INK4a^ negative HPV_16_–DNA-positive	p16^INK4a^ positive HPV–DNA-negative
	*(N* = *620)*	*(N* = *436)*	*(N* = *184)*		*(N* = *29)*	*(N* = *39)*
Risk factors	N (%)*	*N (%)*	*N (%)*		*N (%)*	*N (%)*
Gender						
Male	478 (77)	340 (78)	138 (75)	0.420	23 (79)	32 (82)
Female	142 (23)	96 (22)	46 (25)		6 (21)	7 (18)
Age (IQR)						
Median	60.8	60.0	62.6	**0.002**^**§**^	57.9	60.0
Third quartile	67.8	66.4	71.6		65.2	64.9
First quartile	53.9	53.8	54.1		53.4	56.0
Comorbidity (ECOG)						
0	38 (6)	21 (5)	17 (9)	**0.008**^**#**^	4 (14)	2 (5)
1	398 (65)	277 (64)	121 (67)		17 (61)	26 (67)
2	152 (25)	114 (26)	38 (21)		4 (14)	9 (23)
3	22 (4)	17 (4)	5 (3)		3 (11)	2 (5)
4	5 (1)	5 (1)	0 (0)		0 (0)	0 (0)
Unknown	5	2	3		1	0
Healthy (0–1)	436 (71)	298 (69)	138 (76)	0.059	21 (75)	28 (72)
Sick (2–4)	179 (29)	136 (31)	43 (24)		7 (25)	11 (28)
Alcohol						
>2 standard drinks/day	292 (53)	269 (68)	23 (15)	**<0.001**	20 (77)	15 (44)
≤2 standard drinks/ day	260 (47)	125 (32)	135 (85)		6 (23)	19 (56)
Unknown	68	42	26		3	5
Smoking						
Yes	489 (81)	392 (92)	97 (56)	**<0.001**	26 (90)	31 (82)
No	112 (19)	36 (8)	76 (44)		3 (10)	7 (18)
Unknown	19	8	11		0	1
Pack years (IQR)						
Median	38.0	40.0	25.5	**<0.001**^**§**^	40.0	33.0
Third quartile	50.0	52.5	40.0		61.5	43.5
First quartile	25.0	29.8	15.0		30.0	25.5
Smokers (*N*), pack year unspecified	76	60	16		5	4
Tumour characteristics						
Localisation						
Tonsil	259 (42)	150 (34)	109 (59)	**<0.001**	12 (41)	17 (44)
Other than tonsil	361 (58)	286 (66)	75 (41)		17 (59)	22 (56)
T stage						
1–2	319 (52)	202 (47)	117 (64)	**<0.001**	10 (34)	23 (59)
3–4	297 (48)	230 (53)	67 (36)		19 (66)	16 (41)
1	137 (22)	93 (22)	44 (24)	**<0.001**^**#**^	8 (28)	8 (21)
2	182 (30)	109 (25)	73 (40)		2 (7)	15 (38)
3	128 (21)	98 (23)	30 (16)		9 (31)	7 (18)
4	36 (6)	24 (6)	12 (7)		0 (0)	1 (3)
4a	56 (9)	41 (9)	15 (8)		6 (21)	3 (8)
4b	77 (13)	67 (16)	10 (5)		4 (14)	5 (13)
Unknown	4	4	0		0	0
N stage						
N0	157 (26)	134 (31)	23 (13)	**<0.001**	11 (38)	7 (18)
N+	455 (74)	295 (69)	160 (87)		18 (62)	31 (82)
N0	157 (26)	134 (31)	23 (13)	0.630^#^	11 (38)	7 (18)
N1	106 (17)	53 (12)	53 (29)		1 (3)	6 (16)
N2–N2a	62 (10)	34 (8)	28 (15)		2 (7)	3 (8)
N2b	187 (31)	129 (30)	58 (32)		8 (28)	14 (37)
N2c	75 (12)	59 (14)	16 (9)		4 (14)	7 (18)
N3	25 (4)	20 (5)	5 (3)		3 (10)	1 (3)
Unknown	8	7	1		0	1
M stage						
M0	561 (93)	396 (93)	165 (93)	0.985	25 (89)	35 (92)
M+	44 (7)	31 (7)	13 (7)		3 (11)	3 (8)
Unknown	15	9	6		1	1
UICC7 stages						
I–III	218 (36)	152 (35)	66 (36)	0.881	10 (34)	13 (33)
>III	394 (64)	277 (65)	117 (64)		19 (66)	26 (67)
I	52 (8)	47 (11)	5 (3)	0.277^#^	7 (24)	1 (3)
II	53 (9)	43 (10)	10 (5)		1 (3)	6 (15)
III	113 (18)	62 (14)	51 (28)		2 (7)	6 (15)
IV–IVa	268 (44)	177 (41)	91 (50)		12 (41)	18 (46)
IVb	83 (14)	71 (17)	12 (7)		4 (14)	5 (13)
IVc	43 (7)	29 (7)	14 (8)		3 (10)	3 (8)
Unknown	8	7	1		0	0
UICC-8 stages						
I–III	306 (54)	149 (37)	157 (91)	**<0.001**	12 (43)	12 (32)
>III	265 (46)	249 (63)	16 (9)		16 (57)	25 (68)
I	123 (22)	45 (11)	78 (45)	**<0.001**^**#**^	8 (29)	6 (16)
II	90 (16)	43 (11)	47 (27)		1 (4)	4 (11)
III	93 (16)	61 (15)	32 (18)		3 (11)	2 (5)
IV–IVa	174 (30)	158 (40)	16 (9)		11 (39)	18 (49)
IVb	70 (12)	70 (18)	0 (0)		3 (11)	5 (14)
IVc	21 (4)	21 (5)	0 (0)		2 (7)	2 (5)
Unknown	49	38	11		1	2
Palliative treatment	43 (7)	35 (8)	8 (5)	0.091	2 (7)	0
Curative treatment type						
Upfront surgery without neck dissection	39 (7)	31 (8)	8 (5)	**0.022**	5 (17)	1 (3)
Upfront surgery with neck dissection	325 (54)	210 (54)	115 (68)		17 (59)	20 (53)
Definitive radiotherapy	25 (4)	20 (5)	5 (3)		0 (0)	2 (5)
Definitive chemo–radiotherapy	167 (28)	126 (33)	41 (24)		5 (17)	15 (39)
Unknown	21	14	7		0	1
Resection status of surgery						
R0	268 (79)	177 (79)	91 (81)	0.690	17 (77)	17 (81)
R+	70 (21)	48 (21)	22 (19)		5 (23)	4 (19)
Unknown	26	16	10		0	0

OPSCC patients with discordant HPV–DNA and p16^INK4a^ tests (*N* = 68) are indicated for comparison.

*Percentage based on total cases (*N* = 620) without missing values. *P* values for comparison of HPV-negative versus HPV_16_-driven OPSCC (asymptotic, two-sided) calculated by chi square, (§) *t* test or (#) Mantel–Haenszel test of trend; significant *P* values (*P* ≤ 0.05) in bold.

### HPV status of OPSCC

**Fig. 1:**
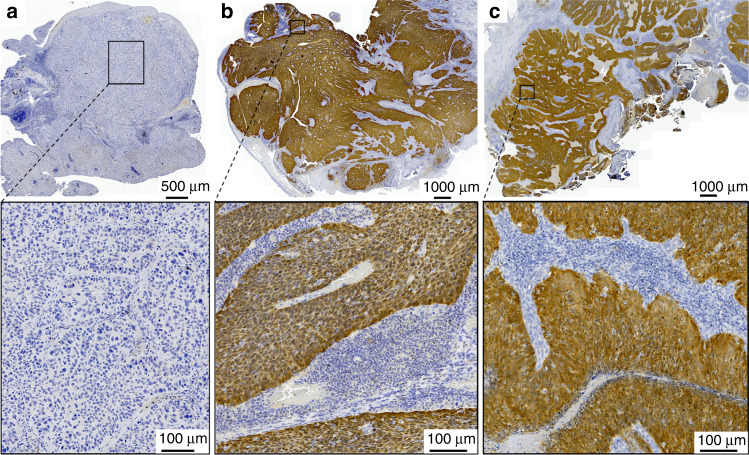
Expression of p16^INK4a^ in oropharyngeal squamous cell carcinomas (OPSCC) detected by immunostaining. **a** HPV–DNA-negative OPSCC without p16^INK4a^ expression. **b** HPV_16_-DNA-positive OPSCC showing strong, diffuse overexpression of p16^INK4a^ characteristic for HPV_16_-driven OPSCC. **c** HPV–DNA-negative OPSCC displaying p16^INK4a^ overexpression similar to HPV_16_-driven OPSCC (**b**).

We have analysed all primary tumours with sufficient formalin-fixed, paraffin-embedded tissue for the presence of HPV–DNA, HPV genotypes (*n* = 721) and expression of p16^INK4a^ (*n* = 717) by immunostaining (Fig. 1) as previously described.^2^ Tumour tissue from 709 (88.4%) diagnostic biopsies (in the case of non-surgical treatment) and resected OPSCC was available to perform both tests.

### Statistics

We used principal component analysis (PCA)^20,21^ as an eigenvector-based multivariate method to reveal the internal structure of our data, especially regarding the four combinations of HPV–DNA testing and p16^INK4a^ expression of OPSCC to define HPV status (HPV-negative and HPV-driven for concordant tests vs. discordant test results). Overall survival (OS, calculated from the date of histological diagnosis by routine biopsy to the date of death from any cause or date of last seen alive) was used to generate survival curves by the Kaplan–Meier method. SPSS Statistical Software (IBM SPSS 26.0) was used for statistical analysis, and significance was considered *P* ≤ .05 for all tests, unless otherwise indicated.

## Results

### HPV types in relation to p16^INK4a^ expression

We detected HR–HPV–DNA in 234/721 (32.5%) OPSCC samples. DNA from non-HR–HPV types was detected in 5 (0.7%) OPSCC only, two of them corresponding to the low-risk (LR) types 6 and 11 (Table 2). HPV_16_ was the most frequent among all HPV types, identified as the exclusive type in 91.2% (and in 94.6% overall) of HPV–DNA-positive cases. HR–HPV types other than 16 were detected alone or in combination with one or two other types in 16 cases (Table 2).

**Table 2:** Prevalence of HPV-driven OPSCC determined by HPV–DNA detection and p16^INK4a^ expression and total frequency of HPV types 2000–2017.

	*n*	*%*	p16^INK4a^ overexpression					
			Negative		Positive		Unknown	
			*n*	*%*	*n*	*%*	*n*	*%*
All OPSCC	802	100.0*	480	59.9*	237	29.6*	85	10.6*
HPV–DNA-negative	482	66.9	**436**	61.5	**39**	5.5	7	
HR–HPV–DNA-positive	234	32.5	37	5.2	192	27.1	5	
Non-HR–HPV–DNA-positive	5	0.7	1	0.1	4	0.6	0	
Unknown	81	10.1*	6		2		73	
High-risk (HR) HPV types								
Single HR type								
16	218	91.2	**29**	13.3	**184**	84.4	5	2.3
18	2	0.8	–	–	2	100.0	–	–
33	1	0.4	–	–	1	100.0	–	–
51	1	0.4	1	100.0	–	–	–	–
58	2	0.8	–	–	2	100.0	–	–
HR double								
16, 18	6	2.5	5	83.3	1	16.7	–	–
16, 53	1	0.4	1	100.0	–	–	–	–
35, 26	2	0.8	–	–	2	100.0	–	–
HR triple								
16, 18, 53	1	0.4	1	100.0	–	–	–	–
Non-HR–HPV types								
Putative HR type								
26	1	0.4	–	–	1	100.0	–	–
Unspecified risk type								
30	2	0.8	–	–	2	100.0	–	–
HPV low-risk type								
6	1	0.4	–	–	1	100.0	–	–
11	1	0.4	1	100.0	–	–	–	–

*Percentage based on total cases (*n* = 802). Bold numbers: patient groups further analysed.

About 27.1% of 709 OPSCC patients were positive for both p16^INK4a^ and HR–HPV–DNA, representing the average prevalence of truly HPV-driven OPSCC during the study period of 18 years. About 61.5% of the samples showed no expression of p16^INK4a^ and did not contain any HPV–DNA, thus being considered truly HPV-negative. Discordant results were observed in 10.7% of all samples analysed for both markers: 5.2% of samples were HPV–DNA-positive but lacked overexpression of p16^INK4a^, and 5.5% of cases displayed p16^INK4a^ overexpression but no HR–HPV–DNA (Table 2).

p16^INK4a^ overexpression was accompanied by positivity for HR–HPV–DNA in 81.7% (192/235) and specifically for HPV_16_–DNA in 78.7% (185/235) of cases. Importantly, in 39 (16.6%) OPSCC overexpressing p16^INK4a^, no HPV–DNA was detected. p16^INK4a^ expression pattern in these HPV– DNA-negative tumours did not differ from HPV-driven OPSCC (Fig. 1). Only OPSCC positive for HPV_16_ or negative for any HPV–DNA (Table 2: highlighted in bold) were included in the descriptive analysis (Table 1).

### Descriptive analysis of OPSCC according to HPV_16_ status

We analysed tumour characteristics, lifestyle- and patient-related risk factors for patients with “truly” HPV-negative (HPV–DNA-negative and p16^INK4a^-negative) and HPV_16_-driven (HPV_16_–DNA-positive and p16^INK4a^-positive) OPSCC. Cases with discordant results were not considered in the statistical comparison of this sub-analysis (Table 1).

**Fig. 2:**
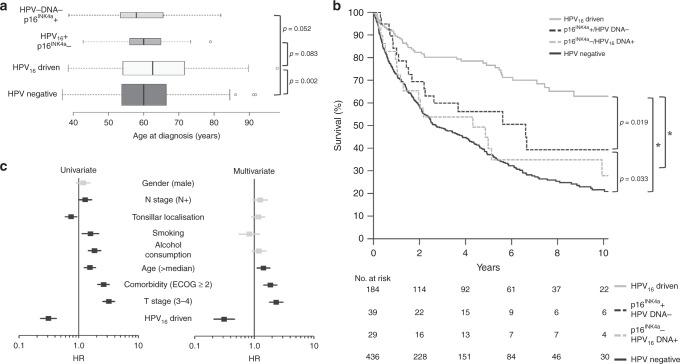
Age at diagnosis in comparison with HPV status and influence of HPV status and risk factors on overall survival (OS) of patients with OPSCC. **a** Age of patients with HPV_16_-driven compared with patients with HPV-negative OPSCC and OPSCC with discordant markers for HPV (HPV_16_–DNA and p16^INK4a^). *p*: *P* value, t test, two-sided; widths of boxes are proportional to square roots of the sample size. **b** OS according to HPV status (including cases with discordant HPV tests). Significant differences in OS are indicated. *p*: *P* value, log-rank test; *: *p* < 0.001. **c** Univariate and multivariate analysis (Cox regression) of risk factors for OS of patients with concordant HPV tests (total: *n* = 620, HPV-negative: *n* = 436, HPV_16_-driven: *n* = 184). Boxes: hazard ratio (HR); horizontal lines: 95% confidence interval; black boxes/lines indicate statistical significance of risk factors.

In contrast to most demographic characteristics and risk factors of OPSCC patients (Table 1), which are in line with the literature, patients with HPV_16_-driven OPSCC were older at diagnosis than patients with HPV-negative OPSCC. The difference in median age was 2.6 years and statistically significant (*P* = 0.002, Fig. 2a, Table 1).

The distribution of treatment modalities significantly differed between patients with HPV_16_-driven and HPV-negative OPSCC, with surgical tumour resection, including neck dissection performed more frequently in patients with HPV_16_-driven OPSCC (Table 1).

### Uni- and multivariate OS analysis of OPSCC according to HPV status

Patients with HPV_16_-driven OPSCC had remarkably better overall survival (OS) than all other patient groups with HPV-negative OPSCC or discordant results for HPV–DNA and p16^INK4a^ expression (*P* ≤ 0.019, Fig. 2b).

In univariate analysis of patients with concordant HPV–DNA and p16^INK4a^ results (HPV_16_-driven and HPV-negative OPSCC), dichotomised variables for age, localisation, comorbidity, tobacco and alcohol consumption and T- and N-stage showed significant impact on patients’ overall survival (Fig. 2c, Supplementary Table S1). Hazard reduction by positive HPV status and low T stage was similar (3.2-fold, *P* < 0.001) and the highest in comparison with all other positive predictors (Supplementary Table S1).

We performed a multivariate Cox regression analysis of OS for patients with concordant markers for HPV status, including all dichotomised variables with significance in the univariate analysis. Age, HPV status, T stage and comorbidity remained independent factors for OS in this multivariate model (*P* < 0.005 each). Again, a 3.2-fold reduction of hazard in the case of HPV_16_-driven OPSCC had the strongest impact on survival (Fig. 2c, Supplementary Table S2).

### Principal component analysis (PCA)

**Fig. 3:**
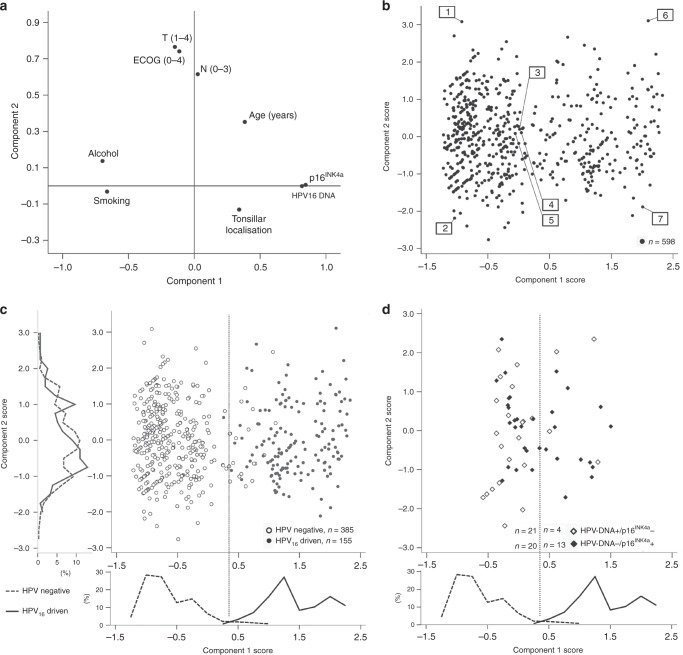
Principal component analysis (PCA) of tumour characteristics and lifestyle/patient-related risk factors and distribution of cases according to the resulting two main components (components 1 and 2). **a** Loading plot of components 1 and 2 using the indicated variables (further details: Table 1). **b** Distribution of all cases (HPV_16_-driven, HPV-negative and discordant HPV tests) without missing data (*n* = 598) according to the resulting main component scores. Selected cases mentioned in detail in Supplementary Table S4 are labelled. **c** Component-1 score efficiently separates HPV_16_-driven and HPV-negative OPSCCs as indicated by the distribution plots next to the axis for both components. A separation border (dashed line) was defined at the central minimum. **d** Applying the separation border defined in (**c**) to cases with discordant HPV tests. The distribution plot of component 1 for HPV_16_-driven and HPV-negative OPSCCs is indicated for comparison.

Patients with discordant HPV-test results differed from those with HPV-negative and HPV_16_-driven OPSCC regarding OS (Fig. 2b), tumour characteristics and lifestyle/patient-related risk factors (Supplementary Table S3). In this multivariate analysis, we included all patients (with complete datasets) and factors significant in univariate analysis, and reduced data dimensionality by principal component analysis (PCA) to two main components. The contribution of each included factor in both components is shown in Fig. 3a. Three clusters of factors with similar impact on the two components with respect to size and direction became evident: 1: p16^INK4a^ expression and HPV_16_ –DNA detection, 2: alcohol consumption and smoking and 3: tumour size (T-) and lymph node (N-) stage together with patient’s performance (ECOG). Age and tonsillar localisation of the primary tumour did not cluster together with other factors.

The distribution of all OPSCCs plotted according to the two main components separated two main groups of patients (Fig. 3b). Risk profiles of selected cases with central or marginal position are shown in Supplementary Table S4.

HPV-negative and HPV_16_-driven OPSCC were clearly separated upon plotting of the individual cases, and a separation border regarding component 1 could be defined according to the distribution of cases. Eighteen of 385 (4.7%) HPV-negative and only one of 155 (0.6%) HPV_16_-driven OPSCC were located outside their respective clusters (Fig. 3c). Applying this separation to OPSCC with discordant HPV-test results showed that 21 of 25 (84%) OPSCC patients positive for HPV_16_–DNA, but lacking p16^INK4a^ expression, clustered together with HPV-negative OPSCC on the left side of the plot (Fig. 3d). These 21 cases resembled patients with HPV-negative OPSCC with respect to risk factors and OS (Fig. 2b) and were not further investigated.

Notably, 20 of 33 (60.6%, group 1) OPSCC with p16^INK4a^ expression but without detection of any HPV–DNA did not cluster together with HPV_16_-driven OPSCC (Fig. 3d). This resulted in two groups showing significant differences: patients of group 1 were characterised by alcohol consumption, smoking and non-tonsillar localisation of the primary tumour. This contrasts with patients of group 2, who were specified by the absence of tobacco and alcohol consumption and tonsillar localisation of the primary tumour (Supplementary Table S5), and clustered together with HPV_16_-driven OPSCC (Fig. 3).

**Fig. 4:**
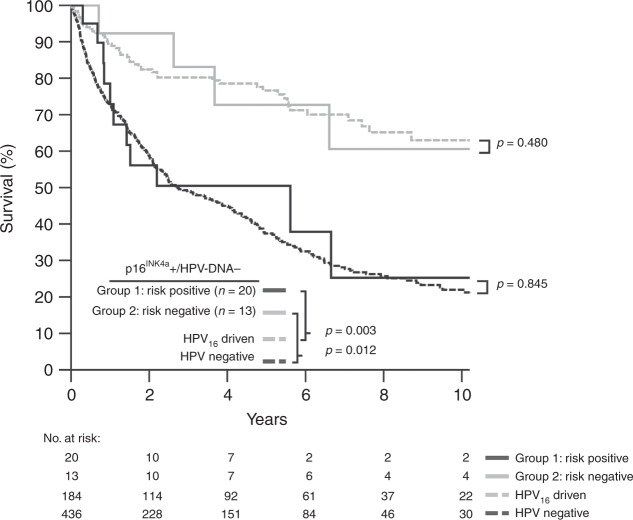
Overall survival (OS) of patients with OPSCC and positive test for p16^INK4a^ expression but lacking detectable HPV–DNA (groups 1 and 2) in comparison with HPV-negative (*n* = 436, dashed black) and HPV_16_-driven (*n* = 184, dashed grey) OPSCC. Patients were stratified applying the separation border defined by PCA (Fig. 3c, d), resulting in patient group 1 (black) with and group 2 (grey) without a more severe risk profile. *p*: *P* value, log-rank test.

Survival analysis revealed that group 1 patients had significantly worse survival compared with patients with HPV_16_-driven OPSCC (Fig. 4, *P* = 0.003), therefore representing “HPV-negative-like” OPSCC. In contrast, no difference in OS existed between group 2 patients in comparison with HPV_16_-driven (*P* = 0.845) but compared with HPV-negative OPSCC (*P* = 0.012, Fig. 4). Treatment of group 1 and 2 patients did not differ in our cohort (Supplementary Table S6). Notably, group 1 patients constituted 10.6% (20/188) of all p16^INK4a^-overexpressing OPSCC in this analysis.

## Discussion

p16^INK4a^ overexpression is currently applied as decision criterion for treatment de-escalation trials in OPSCC.^14,22^ This proceeding relies on the circumstance that p16^INK4a^ overexpression is a surrogate marker of HPV-driven OPSCC, a patient group with a remarkably favourable prognosis. Hence, patients with HPV–driven OPSCC may benefit from de-escalated treatment by the reduction of therapy-related acute and long-term toxicity. This might become relevant for more patients in the future. However, considering the moderate specificity of p16^INK4a^ for HPV-driven OPSCC, concerns have been raised that a subgroup of OPSCC patients could be undertreated in this scenario.^14^ Since robust data estimating the size of this group are presently not available, the major goal of our study was to determine the proportion of these potentially undertreated patients in a large cohort of consecutively characterised patients that allowed the distinction of patient risk-factor profiles in relation to HPV-related markers.

About 30% of OPSCC worldwide are caused by oncogenic HPV types.^1^ We observed an HPV prevalence of 27.1% by simultaneous detection of HR–HPV–DNA and p16^INK4a^ overexpression in our cohort. This represents an average in a central region in Germany over the past 18 years, which is comparable to the global burden. However, we recently showed significant increase of incidence rates in Germany.^2^ In comparison with cervical cancers,^23^ a much higher contribution of HPV_16_ and HPV_18_ is reported for anal (87%) and head and neck (85%) cancers worldwide.^1^ In our study, HPV_16_ was almost the only relevant HPV type for OPSCC. Therefore, we excluded non-16 HPV types from our further investigation.

To our surprise, patients with HPV_16_-driven OPSCC were not younger, but significantly older than patients with HPV-negative OPSCC in our cohort. This is in contrast to the literature, although most published data rely on selected populations from clinical trials,^5,6^ comparatively small random cohorts or cancer registry-based data without inclusion of any experimental data regarding HPV.^24^ Interestingly, the unimodal age incidence distribution was peaking in the United States at ages 60–64 for all patients with HPV-positive OPSCC in a recent investigation,^25^ which is comparable to our data, and more importantly, not younger than HPV-negative OPSCC in our cohort.

However, other lifestyle-related risk factors were obviously reduced in these patients. Furthermore, the type of therapy differed in our cohort according to HPV status, which might be a direct consequence of patient (comorbidity) and tumour characteristics (primary size and N-/M-status) and, therefore, contributes to superior outcome of patients with HPV_16_-driven cancers. Age, HPV status, T stage and comorbidity remained independent risk factors in the Cox regression model. In contrast to other studies,^6^ tobacco and alcohol consumption did not have significant impact on this model. However, both are highly related with HPV status, and in a previous study we showed that tobacco and alcohol consumption were less important for survival prediction in our patients.^26^ In contrast to other studies,^6^ the frequency of smokers in our cohort was higher in general, and especially in HPV-negative OPSCC, which might cover its influence in our multivariate Cox model. This is supported by studies showing that HPV positivity in OPSCC is a significant prognostic factor, irrespective of smoking.^27^

In the PCA, variables for HPV_16_–DNA and p16^INK4a^ had almost similar values of components 1 and 2, and were accompanied by smoking and alcohol consumption on the component-1 axis (Fig. 3a). In accordance with the literature, this demonstrates that p16^INK4a^ overexpression is highly related to the presence of HPV_16_–DNA, and both are negatively related with smoking and alcohol consumption. By geometrical projecting in the PCA, data are transformed onto lower, uncorrelated dimensions with the goal that the first dimension (component 1) explains as much of the data as possible. The second dimension (component 2) explains as much as possible of the remaining, unexplained data (and so on), which reduces complexity by summarising the most contributing features of the dataset. In conclusion, HPV status, smoking and alcohol consumption explained most of the variance in our data by contributing (positively and negatively) to component 1, followed by patient’s performance (ECOG), T- and N-category as they have similar values contributing to component 2 (Fig. 3a). Age and tonsillar localisation were somewhere in-between the aforementioned clusters of factors (Fig. 3a), possibly explained by a closer relation to HPV status (supported by our descriptive data, Table 1 and Fig. 2a). Overall, clustering of the factors fits well to published risk models in OPSCC^6,26,28^ regarding the fact that HPV status is most important followed by ECOG, T- and N-stage. Smoking and alcohol consumption were not the highest in ranking because their association with HPV status most probably covers their influence.

Component-1 values efficiently separated cases with HPV_16_-driven and -negative OPSCC in the PCA (Fig. 3c). The distribution of cases with discordant HPV tests showed that those with HPV_16_–DNA-positive but p16^INK4a^-negative OPSCC resembled HPV-negative OPSCC (except 4/25, 16%), which is supported by similar OS of these patients. In contrast, only 39% (13/33) of OPSCC with p16^INK4a^ overexpression in the absence of any HPV–DNA resembled HPV-driven OPSCC. The remaining patients were similar to patients with HPV-negative OPSCC (notably also with respect to OS) and constituted 10.6% (20/188) of all p16^INK4a^-overexpressing OPSCC included in this analysis. Consequently, if the selection of patients for therapy de-escalation solely relies on p16^INK4a^ expression as an indicator of viral aetiology and improved survival, those patients would be incorrectly selected and thus be put at jeopardy for treatment failure. Our findings clearly show that patient selection for de-escalated treatments based on a single marker bears a substantial risk for OPSCC patients, particularly in populations with high tobacco consumption.

### Summary and conclusion

We have analysed a consecutive cohort of patients and a relevant number of samples lacked either HPV–DNA or p16^INK4a^ positivity. HPV_16_ is by far the most important HPV type in OPSCC in central Germany. Patients with HPV_16_-driven OPSCC consumed fewer amounts of cigarettes and alcohol, but were significantly older at diagnosis. OPSCC positive for p16^INK4a^ but negative for any HPV– DNA, accounting for 10.6% of all p16^INK4a^-overexpressing OPSCC, resembled HPV-negative OPSCC in risk-factor profile and overall survival.

In conclusion, our data imply that p16^INK4a^ expression as a single marker is insufficient to define HPV aetiology in OPSCC, and that stratification of patients for treatment de-escalation thus requires the integration of further risk factors and/or diagnostic tests, primarily confirmatory HPV testing.

## Supplementary information

Supplemental Tables S1–S6

## Data Availability

Data described in this study are available, upon request, from the corresponding author for academic research within the constraints of the consent given by the patients.
