# Correction: Evaluation of p16^INK4a^ expression as a single marker to select patients with HPV-driven oropharyngeal cancers for treatment de-escalation

**DOI:** 10.1038/s41416-021-01457-z

**Published:** 2021-06-10

**Authors:** Steffen Wagner, Elena-Sophie Prigge, Nora Wuerdemann, Henrike Reder, Ayman Bushnak, Shachi Jenny Sharma, Theresa Obermueller, Magnus von Knebel Doeberitz, Thomas Dreyer, Stefan Gattenlöhner, Gregor Wolf, Jörn Pons-Kühnemann, Claus Wittekindt, Jens Peter Klussmann

**Affiliations:** 1 Department of Otorhinolaryngology, Head and Neck Surgery, University of Giessen, Giessen, Germany; 2 Department of Applied Tumor Biology, Institute of Pathology, University Hospital Heidelberg, Heidelberg, Germany; 3 Clinical Cooperation Unit Applied Tumor Biology, German Cancer Research Center (DKFZ), Heidelberg, Germany; 4 Department of Otorhinolaryngology, Head and Neck Surgery, Medical Faculty, University of Cologne, Cologne, Germany; 5 Institute of Pathology, University of Giessen, Giessen, Germany; 6 Medical Statistics, Institute of Medical Informatics, University of Giessen, Giessen, Germany

**Keywords:** Prognostic markers, Oral cancer, Tumour biomarkers, Tumour virus infections, Cancer therapy

Correction to: *British Journal of Cancer* **123**:1114–1122 (2020); 10.1038/s41416-020-0964-x, published online 6 July 2020

The article Evaluation of p16INK4a expression as a single marker to select patients with HPV-driven oropharyngeal cancers for treatment de-escalation, written by Steffen Wagner, Elena-Sophie Prigge, Nora Wuerdemann, Henrike Reder, Ayman Bushnak, Shachi Jenny Sharma, Theresa Obermueller, Magnus von Knebel Doeberitz, Thomas Dreyer, Stefan Gattenlöhner, Gregor Wolf, Jörn Pons-Kühnemann, Claus Wittekindt and Jens Peter Klussmann, was originally published electronically on the publisher’s internet portal on 6 July 2020 without open access. With the author(s)’ decision to opt for Open Choice the copyright of the article changed on 28 May 2021 to © The Author(s) 2021 and the article is forthwith distributed under a Creative Commons Attribution 4.0 International License, which permits use, sharing, adaptation, distribution and reproduction in any medium or format, as long as you give appropriate credit to the original author(s) and the source, provide a link to the Creative Commons licence, and indicate if changes were made. The images or other third party material in this article are included in the article’s Creative Commons licence, unless indicated otherwise in a credit line to the material. If material is not included in the article’s Creative Commons licence and your intended use is not permitted by statutory regulation or exceeds the permitted use, you will need to obtain permission directly from the copyright holder. To view a copy of this licence, visit http://creativecommons.org/licenses/by/4.0/.

Open Access funding enabled and organized by Projekt DEAL.

