# Supplementary material for: Evaluation of p16INK4a expression as a single marker to select patients with HPV-driven oropharyngeal cancers for treatment de-escalation
Source: Br J Cancer. 2020 Jul 6;123(7):1114–22. doi: 10.1038/s41416-020-0964-x (PMC7525437; doi:10.1038/s41416-020-0964-x)
Supplement: Supplementary file 1 — Supplemental Tables S1–S6 [file 41416_2020_964_MOESM1_ESM.pdf]

Supplemental Table S1: Prognostic value of risk factors and tumor characteristics analyzed by univariate survival analyses and by univariate Cox regression.

			univariate survival analysis				Cox regression (univariate)			
		n	Median OS [years]	S.d.	5-year OS *(%)*	*P* [log-rank]	**HR**	95% CI lower	95% CI upper	***P*-value**
**All**		620	4.76	0.39	*(48)*					
**Gender**	Female	142	5.32	0.85	*(54)*	0.194	1			0.195
	Male	478	4.37	0.54	*(46)*		1.189	0.915	1.544	
**Age**	< Median	310	5.88	0.69	*(54)*	**< 0.001**	1			**< 0.001**
	> Median	310	3.55	0.65	*(41)*		1.537	1.242	1.904	
**HPV_{16}-status**	Negative	436	2.71	0.41	*(37)*	**< 0.001**	1			**< 0.001**
	Positive	184	n.d.	n.d.	*(77)*		0.311	0.229	0.420	
**T-stage**	1-2	319	8.71	1.44	*(66)*	**< 0.001**	1			**< 0.001**
	3-4	297	1.65	0.15	*(28)*		3.170	2.539	3.958	
**N-stage**	N0	157	5.54	0.69	*(54)*	**0.046**	1			**0.047**
	N+	455	4.41	0.68	*(47)*		1.285	1.003	1.646	
**Localization**	Non-tonsil	361	4.18	0.56	*(44)*	**0.008**	1			**0.008**
	Tonsil	259	5.88	0.97	*(54)*		0.745	0.598	0.927	
**Alcohol consumption**	No	260	7.63	1.68	*(58)*	**< 0.001**	1			**< 0.001**
	Yes	292	3.06	0.57	*(39)*		1.836	1.454	2.320	
**Smoking**	No	112	10.98	3.48	*(60)*	**0.005**	1			**0.005**
	Yes	489	4.18	0.49	*(45)*		1.573	1.145	2.159	
**Comorbidity (ECOG)**	0-1	436	6.05	0.61	*(57)*	**< 0.001**	1			**< 0.001**
	≥ 2	179	1.38	0.27	*(25)*		2.601	2.089	3.239	

S.d. Standard deviation; n.d. not defined.

Supplemental Table S2: Prognostic value of risk factors and tumor characteristics analyzed by multivariate Cox regression models.

		N	multivariate Cox regression HR	95% CI lower	95% CI upper	***P*-value**	2nd Round HR	2nd Round ***P*-value**	3nd Round HR	3nd Round ***P*-value**	
**All**		620									
**Gender**	Female	142									
	Male	478									
**Age**	< Median	310	1			**0.004**	1	**0.002**	1	**0.001**	
	> Median	310	1.430	1.124	1.821		1.46		1.47		
**HPV$_{16}$-status**	Negative	436	1			**< 0.001**	1	**< 0.001**	1	**< 0.001**	
	Positive	184	0.313	0.212	0.462		0.335		0.320		
**T-stage**	1-2	319	1			**< 0.001[a]**	1	**< 0.001**		**< 0.001**	**T x N**
	3-4	297	2.333	1.808	3.011		2.296		1		
**N-stage**	N0	157	1			0.097[a]	1	0.108			
	N+	455	1.260	0.959	1.655		1.25		2.247		
**Localization**	Non-tonsil	361	1			0.206[b]	1	0.214		0.079	**Localization x Alcohol**
	Tonsil	259	1.172	0.917	1.498		1.168		1		
**Alcohol consumption**	No	260	1			0.178[b]	1	0.195			
	Yes	292	1.201	0.920	1.567		1.190		1.283		
**Smoking**	No	112	1			0.347					
	Yes	489	0.828	0.559	1.229						
**Comorbidity (ECOG)**	0-1	436	1			**< 0.001**	1	**< 0.001**	1	**< 0.001**	
	≥ 2	179	1.862	1.444	2.400		1.851		1.646		

In the second round of the Cox regression, the model did not change substantially by removing the variable for smoking or in the third round, by replacing (a) T-/N-stage, and (b) localization/alcohol consumption, by two respective interaction terms. Still there is a significant influence of the interaction term for T- and N-stage and in case of localization and alcohol consumption, the impact of the interaction term is higher compared to the single variables, although not reaching significance.

Supplemental Table S3: Descriptive analysis of differences in tumor characteristics and lifestyle/patient-related risk factors between OPSCC with concordant and disconcordant HPV-tests (n=688).

statistical difference	*P*	color code
non	*P* > 0.10	(white)
trend	0.10 ≥ *P* > 0.05	(light grey)
significant	**0.05 ≥ *P* > 0.01**	(dark grey)
highly significant	***P* ≤ 0.01**	(black)

OPSCC with concordant HPV-tests			Discordant HPV-tests	
			n=29	n=39
		**HPV-type 16 DNA:**	**positive**	**negative***
		**p16^{INK4a} expression:**	**negative**	**positive**
			P	
n=436 **HPV-negative***	(higher)	**T-stage**	0.588	0.366
	(higher)	**N-stage**	0.470	0.094
	(non-tonsil)	**localization**	0.445	0.250
	(more frequent)	**alcohol consumption**	0.357	**0.004§**
	(more frequent)	**smoking**	0.728	0.070
n=184 **HPV16-driven#**	(lower)	**T-stage**	**0.021**	0.326
	(lower)	**N-stage**	**0.002§**	0.338
	(tonsil)	**localization**	0.071	0.073
	(less frequent)	**alcohol consumption**	**< 0.001§**	**< 0.001§**
	(less frequent)	**smoking**	**0.001§**	**0.004§**

*: negative for all HPV types tested, no p16^{INK4a} expression; #: HPV type 16 DNA positive and p16^{INK4a} overexpression. *P*-values calculated by x^2 test (Pearson, asymptotic, two-sided), significant *P*-values (p≤ 0.05) in bold, §: values remaining significant after adjusting the level of significance according to Bonferroni´s correction for multiple comparisons (*P*≤ 0.01 for n=5 tests).

Supplemental Table S4: Risk profiles of selected cases, to exemplify the influence of risk factors and tumor characteristics in the PCA.

	**1**	**2**	**3**	**4**	**5**	**6**	**7**
HPV16-DNA	no	no	no	no	no	yes	yes
$p16^{INK4a}$ expression	no	no	no	yes	yes	yes	yes
Age	63.5	50.9	67.7	42.9	66.8	88.1	63.4
Comorbidity (ECOG)	4	0	1	2	1	3	0
Alcohol	yes	yes	yes	no	yes	no	no
Smoking	yes	yes	yes	yes	yes	no	no
Tonsillar Localization	no	no	yes	no	no	no	yes
T-stage	T4	T1	T3	T2	T2	T4	T1
N-stage	N2	N0	N2	N2	N2	N2	N0

Blackening of cell filling indicates adverse impact of risk factors and tumor characteristics.

In brief, cases 1 and 2 located to the left in Figure 3B had HPV-negative, non-tonsillar OPSCC with alcohol consumption and smoking history. Higher age, comorbidity, T- and N-stage in comparison to case 2 shifts case 1 to the top in Figure 3B. Cases 6 and 7 located to the right in Figure 3B had HPV-driven OPSCC, without alcohol consumption and smoking history. Non-tonsillar localization of the primary, higher age, comorbidity, T- and N-stage shifts case 6 to the top in Figure 3B. Cases 3-5 have more inconsistent risk profiles and are located close to the origin of both components of the PCA (Figure 3B).

Supplemental Table S5: Descriptive analysis of biometric data of patients with OPSCC negative for HPV-DNA but positive for $p16^{INK4a}$ expression (n=33).

		OPSCC with $p16^{INK4a}$ expression lacking HPV DNA						
		all		group 1 **risk positive** "HPV-negative-like"		group 2 **risk negative** "HPV-driven-like"		
		N	*(%)**	*N*	*(%)*	*N*	*(%)*	*P*-value
**Risk factors**		33	*(100)*	20	*(61)*	13	*(39)*	
Gender	male	28	*(85)*	17	*(85)*	11	*(85)*	1.000#
	female	5	*(15)*	3	*(15)*	2	*(15)*	
Age	< Median	18	*(55)*	12	*(60)*	6	*(46)*	0.435
	≥ Median	15	*(45)*	8	*(40)*	7	*(54)*	
Comorbidity (ECOG)	healthy (0-1)	24	*(73)*	14	*(70)*	10	*(77)*	1.000#
	sick (2-4)	9	*(27)*	6	*(30)*	3	*(23)*	
Alcohol (standard drinks)	>2 / day	15	*(45)*	15	*(75)*	0	*(0)*	**< 0.001**
	≤ 2 / day	18	*(55)*	5	*(25)*	13	*(100)*	
Smoking	yes	27	*(82)*	20	*(100)*	7	*(54)*	**0.002#**
	no	6	*(18)*	0	*(0)*	6	*(46)*	
**Tumor characteristics**								
Localization	tonsil	14	*(42)*	3	*(15)*	11	*(85)*	**< 0.001**
	other than tonsil	19	*(58)*	17	*(85)*	2	*(15)*	
T-stage	1-2	20	*(61)*	10	*(50)*	10	*(77)*	0.122
	3-4	13	*(39)*	10	*(50)*	3	*(23)*	
N-stage	N0	5	*(15)*	4	*(20)*	1	*(8)*	0.625#
	N+	28	*(85)*	16	*(80)*	12	*(92)*	

* Percentage based on total cases (n=33). *P*-values (asymptotic, 2-sided) calculated by chi square or (#) Fisher´s exact test; significant *P*-values (*P* ≤ 0.05) in bold.

Supplemental Table S6: Descriptive analysis of patient´s therapy with OPSCC expressing p16^{INK4a} but lacking detectable HPV DNA.

		Prediction by PCA				
		group 1 **risk positive,** "HPV-negative like"		group 2 **risk negative,** "HPV-driven-like"		
		N	*(%)*	*N*	*(%)*	*P*-value
**curative therapy**	yes	20	*(61)*	13	*(39)*	
**upfront surgery**	no	11	(55)	3	(23)	0.070
	yes	9	(45)	10	(77)	
**neck dissection**	no	10	(50)	4	(31)	0.275
	yes	10	(50)	9	(69)	
**R0 resection**	no	2	(22)	1	(11)	1.000
	yes	7	(78)	8	(89)	
**chemotherapy**	no	7	(37)	7	(54)	0.341
	yes	12	(63)	6	(46)	
**radiation**	no	2	(11)	0	(0)	0.510*
	yes	17	(89)	12	(100)	
**chemoradiation**	no	7	(37)	7	(54)	0.341
	yes	12	(63)	6	(46)	

P-values (asymptotic, 2-sided) calculated by chi square or (*) Fisher´s exact test.
